# Comparison of conventional autopsy and magnetic resonance imaging in determining the cause of sudden death in the young

**DOI:** 10.1186/1532-429X-16-44

**Published:** 2014-06-19

**Authors:** Rajesh Puranik, Belinda Gray, Helen Lackey, Laura Yeates, Geoffrey Parker, Johan Duflou, Christopher Semsarian

**Affiliations:** 1 Department of Cardiology, Royal Prince Alfred Hospital, Sydney, NSW, Australia; 2 Sydney Medical School, University of Sydney, Sydney, NSW, Australia; 3 Specialist Magnetic Resonance Imaging, Royal Prince Alfred Hospital Medical Centre, Sydney, NSW, Australia; 4 Agnes Ginges Centre for Molecular Cardiology, Centenary Institute, Sydney, NSW, Australia; 5 Department of Radiology, Royal Prince Alfred Hospital, Sydney, NSW, Australia; 6 Department of Forensic Medicine, Sydney, NSW, Australia

## Abstract

**Background:**

Sudden death in the young is a tragic complication of a number of medical diseases. There is limited data regarding the utility of post-mortem Magnetic Resonance (MR) imaging and Computer Tomography (CT) scanning in determining the cause of sudden death. This study sought to compare the accuracy of post-mortem cross-sectional imaging (MR and CT) with the conventional autopsy in determining the cause of sudden death in the young.

**Methods:**

Consecutive patients from 2010 to 2012 (aged 1–35 years) who had sudden death were included. Patients were scanned by CT and 1.5 T MR imaging prior to the conventional autopsy being performed. The primary outcome was diagnostic congruence between imaging and conventional autopsy.

**Results:**

In 17 patients studied, the mean age at death was 23 ± 11 years, with a male predominance (n = 12; 71%). The most common cause of death was a primary cardiac pathology (n = 8; 47%), including ARVC (24%) and ischemic heart disease (12%). Non-cardiac causes identified included pulmonary embolism (6%), and aortic dissection (6%). MR imaging correctly identified the diagnosis in 12 patients who subsequently had positive findings at conventional autopsy, while the diagnosis in the remaining 5 cases remained unexplained. MR imaging was found to be highly sensitive (100%) with a high negative (100%) and positive (80%) predictive value.

**Conclusions:**

Dedicated post-mortem MR imaging of the heart and brain is a useful modality in determining the cause of sudden death in children and young adults, particularly in situations where a conventional autopsy cannot be performed for logistic, cultural or personal reasons.

## Background

Sudden death is defined as death which is unexpected and natural, generally occurring within one hour of onset of symptoms [1]. A significant proportion of sudden deaths are unexplained, with no abnormalities identified at autopsy in approximately 18% (range 6-35%) [2-7]. Amongst sudden deaths attributable to a cardiac cause, structural abnormalities are detected at autopsy in approximately 69% [3]. These pathologies include ischemic heart disease, cardiomyopathy, myocarditis and aortic disease, all of which are routinely detected with cardiac imaging ante-mortem [2-7]. Approximately 30% of sudden cardiac deaths remain unexplained, most of which are presumed to be secondary to cardiac arrhythmias in patients with structurally normal hearts [3,6,8].

Traditional post-mortem examination of the deceased is performed by a trained forensic pathologist, and includes macroscopic and microscopic examination of the main organs, toxicology analysis, and biochemical testing [9,10]. There are a number of issues with the traditional post-mortem examination including emotional trauma for the decedent’s family, cost of the procedure, and delay in delivery of the body to the family for religious rites of death, which is a significant issue for some religions [11]. Using post-mortem imaging as an alternative “minimally invasive” autopsy has been proposed [12]. The majority of studies to date examining the accuracy of post-mortem magnetic resonance (MR) imaging examination have focused on fetal and neonatal deaths [13-17]. A single study comparing post-mortem CT and MR imaging in assessing cause of death in adults suggested CT may be more accurate in this setting [18]. Whilst it seems that high-field MR imaging has a definite role in the diagnosis of sudden natural death in the fetus, the utility in older decedents remains less certain [19].

The current study sought to assess the accuracy of post-mortem CT and MR imaging in determining the cause of death in children and young adults with sudden death, when compared to the gold standard of traditional invasive post-mortem examination.

## Methods

### Patient selection

Deceased patients were selected from those referred from the Sydney metropolitan area to the Department of Forensic Medicine (DFM), in Glebe, NSW, Australia. In this prospective study, consecutive patients from 2010 to 2012, aged between 1–35 years were screened. Those whose death was identified to be sudden were included. Any patients with trauma, suicide or known drug overdose were excluded. Staff at the DFM identified eligible patients upon receipt of the body of the deceased. Patients were then referred to the CT and MR imaging radiographers at the imaging department. Any patient for whom scanning time was available and would not delay time to autopsy by more than 24 hours were included. Verbal and written consent for the study was obtained from the deceased next of kin by trained senior grief counselors and study researchers. Verbal consent was also obtained from the duty coroner. The deceased were then transported to the imaging department in a non-ferrous bag covered with a drape, for scanning. Patients were scanned between 6-8 pm and were returned to the DFM after scanning for the conventional autopsy the following morning.

The circumstances surrounding the death and the deceased’s prior medical history, as obtained by police investigating the death on behalf of the coroner, were made available to the imaging team and to the autopsy pathologist prior to the commencement of their respective examinations. Both the imaging and pathology teams had access to this information. Neither team had access to the findings of the other prior to completion of their respective reports. Following completion of the imaging and autopsy reports, both teams had access to the full reports for comparison purposes. The CT and MR reports were included in the completed death investigation report, and made available to the Coroner and if requested to the next of kin of the deceased and medical practitioners treating the deceased and family members.

The study was conducted with the permission of the Office of the NSW State Coroner, and performed with maintenance of confidentiality, and in accordance with the Sydney Local Health District human ethics guidelines.

### MR imaging protocol

MR imaging was performed on a Philips 1.5 T Achieva MRI system (Philips Medical Systems, Best, The Netherlands). The subject was placed on the scan table in the supine position within the body bag. Brain and cardiac sequences were acquired. The following sequences were acquired for brain scanning: sagittal T1, 3D T1 FFE, axial T2, Dual Echo STIR, coronal T2 FLAIR, FFE and IR. The cardiac sequences acquired included balanced FFE and T2 STIR in the short axis plane through the entire cardiac mass. A T2 STIR sequence was performed in multiple long axis cardiac planes including; LVLA, RVLA, four chamber, LVOT and RVOT. Myocardial oedema was diagnosed when there was increased by signal intensity on T2 weighted imaging. Specifically, significant hyper-intensity was defined when signal intensity was greater than 2 standard deviations of that of remote normal myocardium in the same slice, if this pattern did not correlate with a known coronary artery territory, the oedema was presumed to be secondary to myocarditis by the imaging team. Traditional radiologic planes were acquired, in particular in an axial T1, axial T2, axial T2 STIR and axial balanced FFE. Finally, a 3D whole heart sequence performed through the entire thorax, including the great vessels (Additional file 1: Table S1 MRI techniques). Images were reviewed by a cardiologist (RP, Level III SCMR accredited in Cardiovascular MR) and an experienced senior radiologist (GP) and blinded reports were generated.

### CT protocol

CT scanning was performed on a GE VCT 64-slice scanner (General Electric corporation Milwaukie, USA). As with MR imaging, the subject was placed on the scan table in the supine position within the body bag. Non-contrast scanning was performed in helical mode in a cranial to caudal direction through the head, neck, chest, abdomen and pelvis. Scanning parameters included: 120/140 Kvp, variable Mas (max 700) smart Ma set to noise index of 6 for the brain, 25 for the neck to pelvis and a rotation time of 1 second. Contiguous thin slice prospective reconstructions in the axial plane of 0.625 mm were generated using both soft tissue and bone algorithms. Multi planner images were then generated in the axial and coronal planes through the brain, neck, chest, abdomen and pelvis of a thickness of 5 mm. Thin slice data was copied onto disc for storage/review or any required reworking. Subsequently, data was reviewed by the senior radiologist (GP) and a report was generated. Both the CT and MR imaging reporters were blinded to the results of the conventional autopsy, and only had access to the autopsy report and results of other post-mortem investigations after their reports had been generated.

### Conventional autopsy

Following post-mortem MR imaging and CT scanning, conventional autopsy was performed in all cases by a trained pathologist (JD). This included toxicology for common drugs and poisons. Histology was performed on all major body organs as per standard protocols. Fixed brain neuropathological examination was performed in cases where the pathologist had a high index of suspicion of neuropathological abnormalities. Identification of pathology in the subject was as per standard autopsy criteria. Blood samples were taken at time of autopsy for molecular genetic testing [9].

### Statistical analysis

Statistical analysis was carried out using Microsoft Excel and GraphPad Prism (Version 6.0) software. Continuous variables were analysed using unpaired t-tests. A p value was considered significant if <0.05. Sensitivity, specificity, positive predictive value and negative predictive value were calculated using standard formulae.

## Results

### Cohort characteristics

There were 17 patients identified from 2010 to 2012 who were appropriate for post-mortem imaging investigation. The baseline characteristics are shown in Table 1. The mean age at death was 22.7 ± 10.8 years (range of 1.5 to 35 years. The majority (n = 12, 71%) of the patients were male. The mean body mass index (excluding the one patient aged 18 months) was 25.6 ± 5.8 kg/m^2^. There was a family history of sudden death in two patients (12%). The details surrounding the circumstances of the deaths are shown in Table 2.

**Table 1:** Baseline characteristics of young sudden death cases (n = 17)

**Age at death (yrs)**	22.7 ± 10.8
**Male (%)**	71%
**BMI (kg/m2)**	25.6 ± 5.8
**Time to autopsy (hrs)**	56.1 ± 17.2
**Family history of sudden death, n (%)**	2 (12)
**Cause of death, n (%)**	17 (100)
1) Primary Cardiac	8 (47)
• ARVC	4 (24)
• CAD	2 (12)
• HCM	1 (6)
• Myocarditis	1 (6)
2) Neurological	2 (12)
• GBM	1 (6)
• SUDEP (Alagille syndrome)	1 (6)
3) Vascular	2 (12)
• Pulmonary embolus	1 (6)
• Aortic dissection	1 (6)
4) Unexplained	5 (35)

NB: BMI = body mass index; ARVC = arrhythmogenic right ventricular cardiomyopathy; CAD = coronary artery disease; HCM = hypertrophic cardiomyopathy; GBM = glioblastoma multiforme; SUDEP = sudden unexpected death in epilepsy.

**Table 2:** Circumstances of sudden death

**Patient**	**Sex/Age (yrs)**	**Circumstances of death**	**Place & time of death**	**Documented arrhythmia**	**Preexisting condition**	**MRI diagnosis**	**CT diagnosis**	**Autopsy diagnosis**
1	F/28	Found semiconscious in bed by sisters, moaned “ambulance” then lost consciousness.	Home 2330 pm	No	Hypertrophic cardiomyopathy, last follow up 5 yrs prior	Hypertrophic cardiomyopathy	Unexplained	Hypertrophic cardiomyopathy
2	M/28	Brother heard loud bang overnight, found deceased on floor next morning.	Home Overnight	No	No	ARVC	Unexplained	ARVC
3	M/35	Complained of chest and arm pain earlier in day. Went to bed as felt unwell later found dead.	Home 0600-1200 pm	No	No	Acute myocardial infarction	Unexplained	Acute myocardial infarction
4	M/29	Found slumped over toilet seat at home, last seen 12 hours prior.	Home 1930 pm	No	Epilepsy, Alagille Syndrome	SUDEP (Grey matter heterotopia)	Unexplained	SUDEP
5	M/31	Went for evening jog (usual for him), found by passers by prone on grass footpath.	Outdoors 1945 pm	No	No	Ruptured aortic aneurysm	Ruptured aortic aneurysm	Ruptured aortic aneurysm
6	M/1.5	NOK put deceased to bed on his back, found short time later cyanosed and not breathing with vomit near mouth	Home 1940 pm	No	Febrile convulsions	Pneumonia	Unexplained	Unexplained
7	M/16	Complained of flu-like symptoms for preceding 24 hours. Went to bed as felt unwell, found by NOK not breathing in bed.	Home 1720 pm	Asystole	No	Hypertrophic cardiomyopathy	Unexplained	Unexplained
8	M/32	Collapsed unconscious while dancing in a salsa club.	Hospital 0000-0600 am	No	Type II DM, hypertension, hypercholesterolaemia	Coronary artery disease	Perforated viscus	Coronary artery disease
9	M/17	Found deceased in bed.	Home Overnight	No	No	ARVC	Unexplained	Unexplained
10	F/35	Collapsed in bathroom, found by husband not breathing.	Home 1900 pm	No	No	ARVC	Pulmonary haemorrhage	ARVC
11	F/17	Found deceased in bed.	Home Overnight	No	No	ARVC	Unexplained	ARVC
12	M/26	Complained of nausea and breathlessness then noted to lose consciousness. Had noticed breathlessness and leg pain previous 3–4 days.	Hospital 0249 am	No	No	Pulmonary embolus	Pulmonary embolus	Pulmonary embolus
13	F/26	Presented to hospital with chest pain, nausea and chills. Rapid deterioration and cardiac arrest	Hospital 0645 am	No	No	Myocarditis	Unexplained	Myocarditis
14	M/4	Diarrhoea and vomiting day prior to death. Then noted to be lethargic, taken to hospital but deteriorated.	Hospital 1735 pm	No	No	Unexplained	Intraabdominal bleed	Unexplained
15	M/27	Found unconscious on roadside whilst out jogging.	Hospital 1200 pm	VF	No	ARVC	Unexplained	ARVC
16	M/5	Found in bed not breathing after vomiting. Had been complaining of headaches for 2 week.	Home 0320 am	No	No	Intracranial tumour	Intracranial tumour	Intracranial tumour
17	M/29	Complained of nausea, went to sleep and found deceased by friends following morning.	Home Overnight	No	No	Unexplained	Unexplained	Unexplained

NB: M = male; F = female; ARVC = arrhythmogenic right ventricular cardiomyopathy; SUDEP = sudden unexplained death in epilepsy; VF = ventricular fibrillation.

### Post-mortem autopsy findings

**Figure 1:**
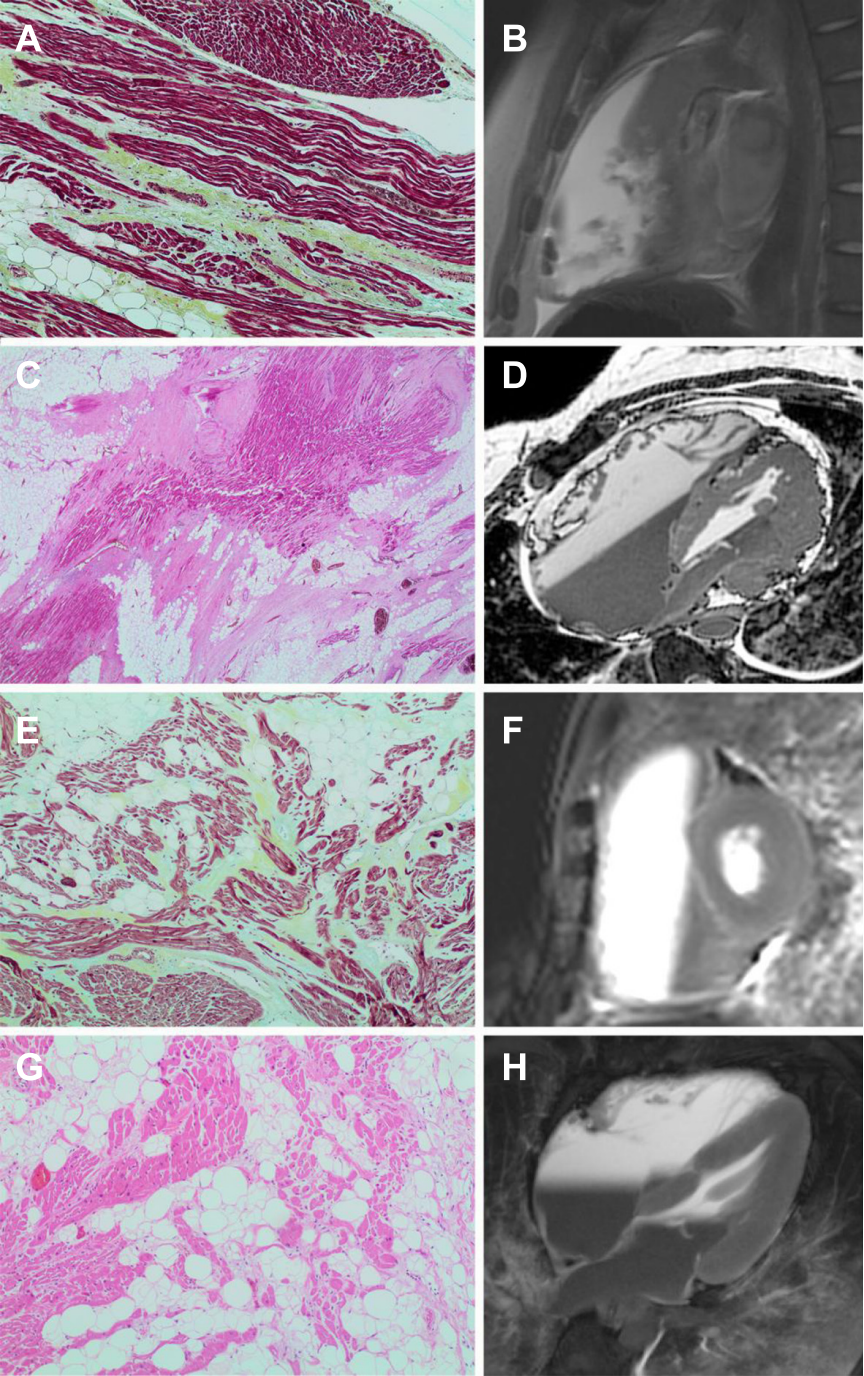
**Post-mortem histopathology with correlating MR images in four patients with ARVC. (A and B)** Patient 2: Right ventricular outflow tract showing established focal fibrosis. Movat pentachrome stain, 10x objective. Sagittal thoracic MR b-FFE image demonstrating marked RVOT dilatation. **(C and D)** Patient 10: Interventricular septum showing extensive fatty infiltration and fibrosis with associated loss of myocytes. H&E stain, 2x objective. Oblique axial 3-d whole heart MR image demonstrating marked RV and RA dilatation. **(E and F)** Patient 11: Right ventricular outflow tract showing established fibrosis and fatty infiltration. Movat pentachrome stain, 10x objective. Basal short axis b-FFE MR image demonstrating severe RV dilatation. **(G and H)** Patient 15: Right ventricular outflow tract showing focal fibrosis, scanty lymphocytic infiltrate and extensive fatty infiltration. H&E stain, 10x objective. 4-chamber b-FFE MR image demonstrating moderate RV dilatation.

**Figure 2:**
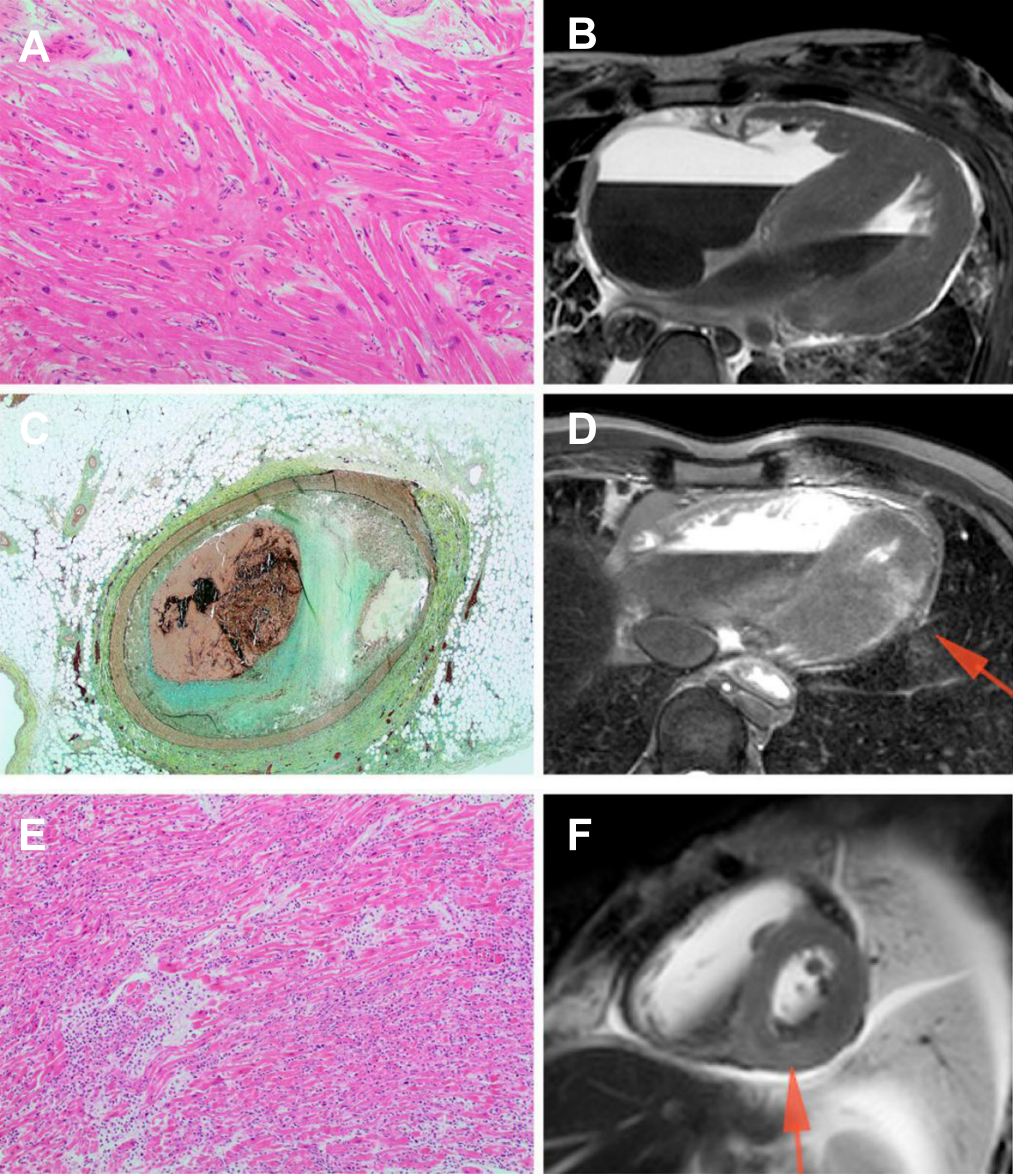
**Post-mortem histopathology with correlating MR images in three patients with other cardiac causes of death. (A and B)** Patient 1 with HCM: Interventricular septum showing myocyte disarray and hypertrophy. H&E stain, 10x objective. 4-chamber b-FFE MR image demonstrating severe basal septal hypertrophy and normal lateral wall thickness. **(C and D)** Patient 3 with acute myocardial infarction. Left anterior descending coronary artery atherosclerosis with acute occlusive thrombus. Movat pentachrome stain, 2x objective. 4-chamber T2 STIR MR image demonstrating regional hyperintense signal in the distal lateral wall, consistent with acute myocardial infarction/oedema. **(E and F)** Patient 13 with myocarditis. Myocardium showing extensive lymphohistiocytic infiltrate with associated myocyte necrosis. H&E stain, 10x objective. Short axis T2 STIR MR image demonstrating diffuse hyperintense signal in the basal infero-septal wall, consistent with myocarditis/myocardial oedema.

**Figure 3:**
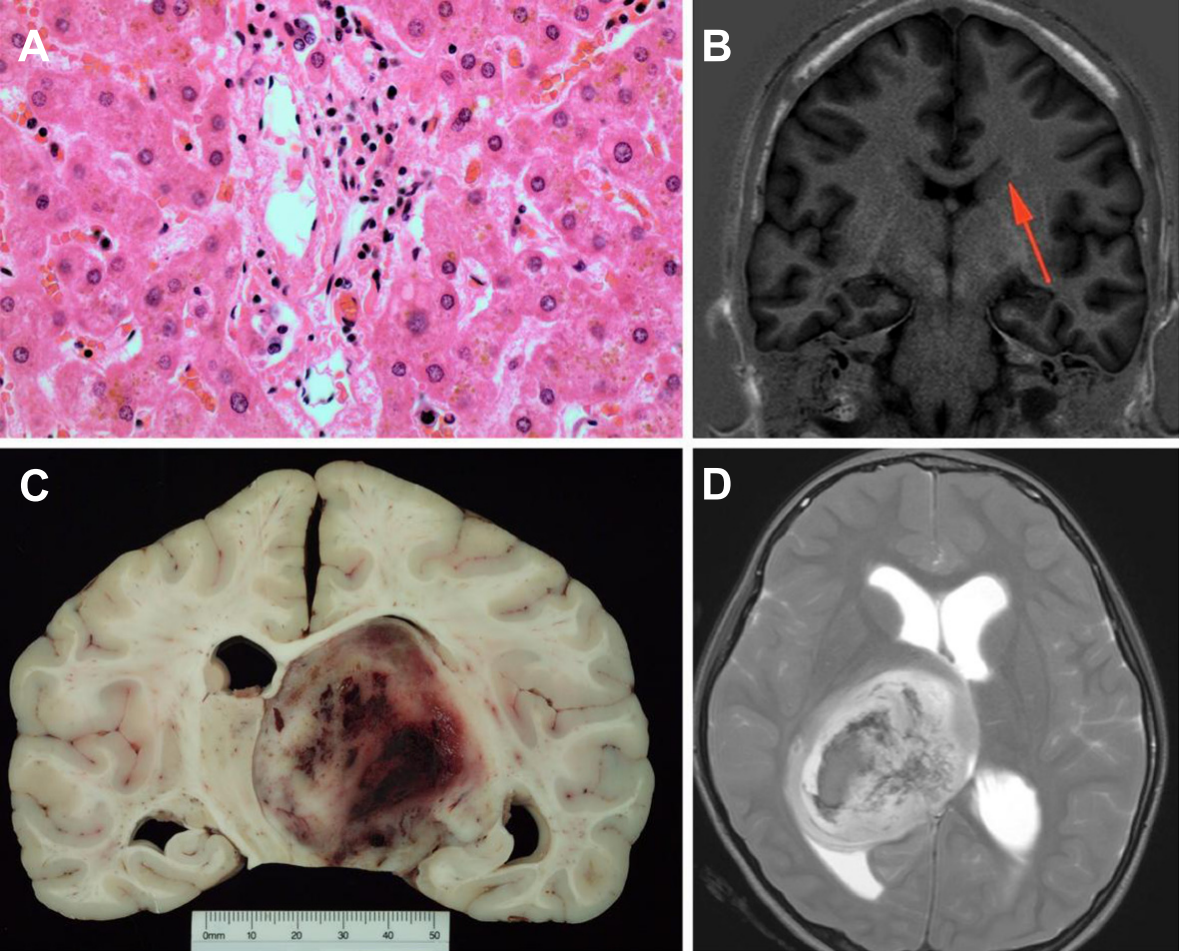
**Post-mortem pathology with correlating MR image in two patients with death secondary to neurological pathology. (A and B)** Patient 4 with Alagille syndrome. Liver showing a relative paucity of bile ducts in portal tract, typical of Alagille syndrome (arteriohepatic dysplasia). H&E stain, 40x objective. Coronal T1 inversion recovery brain MR image demonstrating unusual grey matter signal in temporal lobe white matter, suggesting grey matter heterotopia **(C and D)** Patient 17 with primary brain tumour. Thalamic glioblastoma multiforme, Grade IV, with intratumoral haemorrhage. Sagittal T2 weighted MR Image showing large well-circumscribed mass lesion in right thalamus with associated haemorrhage consistent with vascular tumour

**Figure 4:**
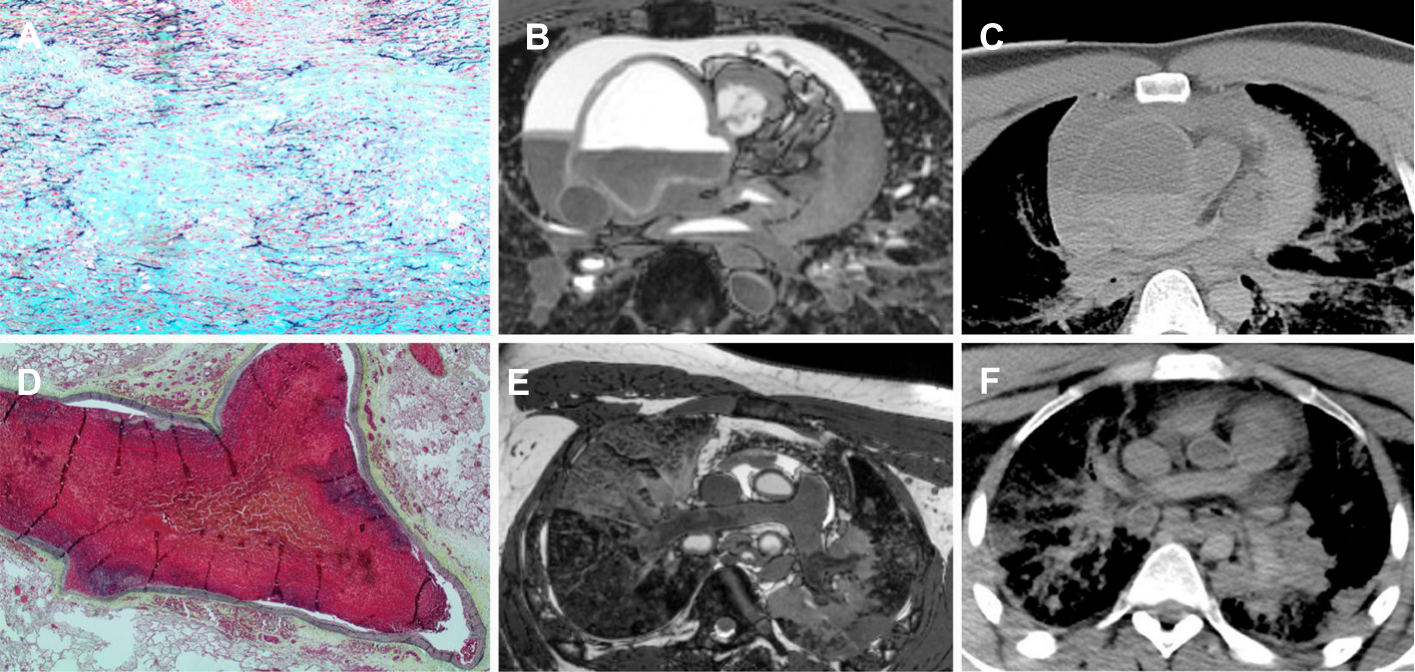
**Post-mortem histopathology with correlating MR image and correlating CT image in two patients with death secondary to primary vascular cause. (A, B and C)** Patient 5 with aortic dissection and cardiac tamponade. Aorta showing marked cystic medial degeneration with large pools of acid mucin. Marked disruption of the elastin fibres is noted. Movat pentachrome stain, 10x objective. MR 3-d whole heart image demonstrating severe ascending aortic dilatation, where there is aortic dissection noted posteriorly and there is an associated large haemopericardium. CT image also demonstrating dilated ascending aorta and haemopericardium. The region of dissection is less well identified due to the lower resolution of this scan when compared to the dedicated MR imaging. **(D, E and F)** Patient 12 with bilateral pulmonary emboli. Occlusive thromboembolism showing early healing by organisation in large pulmonary artery. Movat pentachrome stain, 2x objective. MR 3-d whole heart image demonstrating bilateral pulmonary emboli in the distal branch pulmonary arteries. Note the heterogeneous signal from this region of clot, especially when compared to the more proximal pulmonary arteries, which is more typical of the expected homogenous signal derived from post-mortem related clot. CT showing the branch pulmonary arteries, where clot in the distal RPA is better visualised than in the LPA and the imaging is generally lower resolution than the dedicated MR Imaging.

The mean time to autopsy was 56.1 ± 17.2 hours. The most common cause of death was primary cardiac pathology (n = 8, 47%). Of these half (n = 4, 24%) were found to be due to arrhythmogenic right ventricular cardiomyopathy (ARVC) (Figure 1). Amongst these patients there were pathognomonic histopathological features of ARVC. All had macroscopic evidence of RVOT fat, with one also having obvious RV dilatation. Histologically three of these patients had evidence of interstitial fibrosis, two had replacement fibrosis and all four patients had histological fibro-fatty infiltration.Amongst the four other patients who died secondary to cardiac causes (Figure 2), two patients (12%) died secondary to ischemic heart disease. Both of these patients had evidence of significant coronary artery disease macroscopically. One had evidence of acute thrombus in the left anterior descending artery and one had severe atherosclerosis with evidence of acute plaque rupture. One patient died secondary to hypertrophic cardiomyopathy (HCM). This was a known premorbid diagnosis, however the patient had been lost to follow up for over five years. This patient had macroscopic evidence of severe asymmetric septal hypertrophy (interventricular wall thickness 20 mm) and histologic evidence of myocyte hypertrophy, myofibre disarray, replacement fibrosis and interstitial fibrosis. The final patient had cardiac death secondary to myocarditis, with evidence of macroscopic focal mottling with endocardial discolouration, with microscopic lymphocytic and plasma cell infiltrate with myocardial necrosis.Two patients died from neurological causes (n = 2, 12%). One patient died due to a primary brain tumour (glioblastoma multiforme) and one from sudden unexplained death in epilepsy (SUDEP) in a patient with Alagille syndrome (Figure 3). There were two deaths (12%) secondary to primary vascular causes. One death was due to aortic dissection with cardiac tamponade and one due to bilateral pulmonary emboli (Figure 4). Five of the patients (35%) did not have a cause of death identified at conventional autopsy, with the death being labelled as “unexplained”.

### Comparison of autopsy and imaging findings

**Figure 5:**
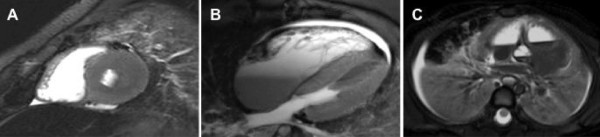
**Post-mortem MRI scans for patients with false positive imaging and unexplained autopsy diagnosis. (A)** Patient 7 with incorrect diagnosis of hypertrophic cardiomyopathy. Short axis STIR showing asymmetrically increased wall thickness in the posterior left ventricular wall. **(B)** Patient 9 with incorrect diagnosis of arrhythmogenic right ventricular cardiomyopathy. 4-chamber STIR showing relatively larger RV size in comparison to LV. **(C)** Patient 6 with incorrect diagnosis of pneumonia. STIR image through lung and heart showing appearance of lung consolidation with associated collapse bilaterally.

Compared to the gold standard traditional post-mortem evaluation, MR imaging correctly identified the pathology in 12 patients. Importantly, there were no false negative results. There were two true negative results from MR imaging as well as three false positives (one incorrect diagnosis of each of HCM, ARVC and pneumonia (Figure 5). MR imaging was found to be highly sensitive (100%) with a high negative predictive value (100%). There was also a high positive predictive value (80%). Specificity of MR imaging was 40% due to the three false positive results. The general accuracy of computer tomography (CT) scanning was lower than with MR imaging. CT scanning only identified the correct pathology in three cases. The sensitivity of post-mortem CT scanning was 33% with a negative predictive value of 45%. The positive predictive value was 50% with a specificity of 63%.

There were five cases in which the cause of death was not identified at post-mortem. Amongst these cases, two patients had negative MR imaging results, and three patients had false positive MR imaging results. Amongst these same five patients, four had negative CT scans with one false positive CT scan.

### Ventricular planimetry on MR imaging

**Figure 6:**
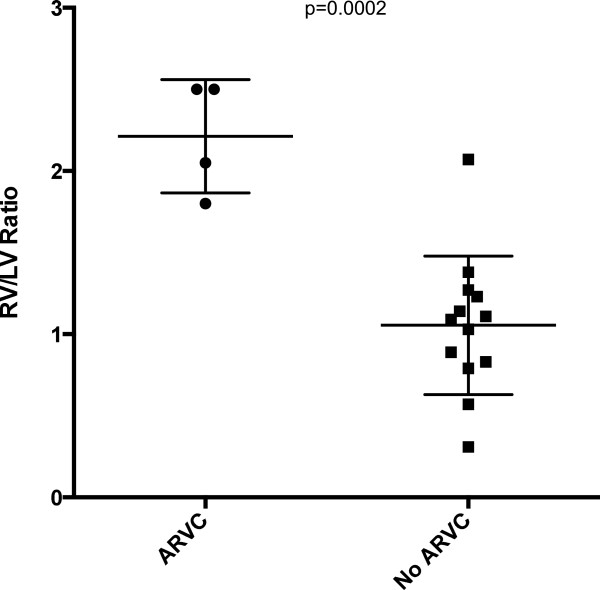
Scatter plot showing significantly larger RV: LV ratio in patients with ARVC (RV: LV= 2.2 ± 0.3) compared to those who did not have a diagnosis of ARVC (RV: LV= 1.1 ± 0.4, p=0.0002).

A significant proportion of the cohort (n = 4, 24%) was found to have ARVC as their cause of death. MR imaging was able to identify these patients with a high degree of accuracy, particularly when using post-mortem ventricular area assessment. The patients who had ARVC as their cause of death were noted to have a significantly higher right ventricular (RV) to left ventricular (LV) area ratio (RV: LV = 2.2 ± 0.3) derived from direct planimetry of the endocardial surface from the 4 chamber STIR image, compared to those who did not have a diagnosis of ARVC (RV: LV = 1.1 ± 0.4, p = 0.0002) (Figure 6).

## Discussion

In this study, the conventional autopsy was compared to CT and MR imaging in establishing the underlying cause of sudden death in the young. When compared to the “gold-standard” of conventional autopsy, a high utility in performing MR imaging in cases of sudden death was demonstrated, where the sensitivity, and positive and negative predictive values were high. However, the specificity of this modality was considerably lower. By comparison, CT had lower sensitivity, positive and negative predictive values but did have a higher specificity. These findings confirm a definite role for brain and cardiac MR imaging in cases of sudden death in the young, where traditional conventional autopsy is not possible, with implications for screening family members of the decedent.

There are relatively few published studies examining the role of post-mortem imaging in children and adults compared with conventional autopsy [13,15,17-22]. In a systematic review performed by Thayyil *et al.*, 35 children and adults had been studied in this way between 1990 and 2009, where the sensitivity was 28% and the specificity was 64% for these studies [22]. The diagnostic yield of MR imaging favoured identifying pathology in fetuses, rather than in older children or adults. In 2012, Roberts *et al.*, presented the largest cohort to date of adult deaths investigated with post-mortem CT and MR imaging and found that CT was the superior modality in this validation study [18]. The investigators reported high discrepancy rates generally between radiology and conventional autopsy, which varied between 32-43% and may in part reflect the difference in CT technology used (16 slice or less) and/or differing cardiac MR imaging protocols performed. Interestingly, common causes of sudden death cases specifically were frequently missed on CT and MR imaging in the study, however the overall impression was that CT and MR imaging may be useful in some circumstances compared with the traditional autopsy [18]. Most recently, a series of 400 post-mortem cases in fetuses and children was reported, which showed high sensitivity, specificity, positive and negative predictive values in identifying structural cardiac pathology post-mortem with cardiac MR imaging [17]. While the cohort differs from our study as they only included fetuses and children ≤16 years, with nearly three quarters of their cohort being stillborn fetuses, the study supports the utility of post-mortem MR imaging as an alternative to conventional autopsy.

In our current study, performed in a centre with a high degree of MR imaging expertise, the findings support the notion that cross-sectional imaging with MR imaging and whole body CT has an important role in the diagnosis of intra- and extra- cardiac and brain pathologies at post-mortem. Importantly, where these structural causes of death are excluded, this allows the focus of the investigation to concentrate on genetic and inherited pathologies, such as channelopathies [5,8,10,23]. The high sensitivity and PPV of MR imaging suggests this modality to be a good “rule out” test in the investigation of sudden death. Our study also supports an important role for MR imaging in the diagnosis of extra-cardiac or brain pathology. Specifically, the diagnosis of great vessel pathology such as aortic rupture and large pulmonary emboli were reliably detected with MR imaging.

Amongst the intra-cardiac group, there was a high detection rate of presumed ARVC, which relied on the presence of global or regional dilatation of the RV in the absence of any other obvious loading conditions, such as pulmonary valve or artery pathology [24]. We suggest that when the RV area is greater than two times the LV area on a 4-chamber MR imaging view of the heart or when there is regional dilatation present in the RVOT, that the diagnosis of ARVC should be considered. It should be noted that our protocols for the heart include multiple long and short axis planes of the heart, along its own oblique axis rather than in conventional radiologic planes, which have not been specifically investigated in previous reports [18,19]. This may allow for greater sensitivity in detecting cardiac pathology post-mortem. Our study showed both MR imaging and CT to be accurate in diagnosis of neurological abnormalities, which is consistent with other earlier studies [13,15].

Importantly, there were no false negative cases in our cohort and three false positive cases. There are important lessons to be learnt, even from this small cohort where caution should be exercised in the post-mortem diagnosis of HCM and lung pathology on MR imaging. Macroscopic lung appearances at post-mortem do not correlate well with histological findings [25], and similarly post-mortem imaging studies of the lungs have been shown to be inaccurate, except in diagnosing major structural abnormalities [19]. Indeed in our cohort, there was an incorrect diagnosis of pneumonia through MR imaging in a patient whose conventional autopsy diagnosis remained unexplained, while a diagnosis of pulmonary embolism was correctly identified by both MR and CT imaging. HCM often presents diagnostic difficulties at post-mortem due to myocardial oedema increasing the apparent wall thickness of the heart, especially at the level of the papillary muscle. In our series, there were two such cases, one where hypertrophy involving the basal septum and MR imaging correctly identified HCM. The other was thought to be an atypical variant of HCM, where hypertrophy affected the mid-posterior wall at the level of the papillary muscle. We recommend that this diagnosis only be made in the basal septal region where there is definite asymmetry and encourage the use of genotyping [26] and histological confirmation of myocardial disarray.

Although CT imaging is more likely to be available for use in most forensic facilities, in the context of sudden death in adults, this modality had disappointingly low levels of diagnostic accuracy. As expected CT imaging was able to identify brain and extra-cardiac pathology similar to MR imaging. However, CT was unable to define accurately any of the intra-cardiac pathologies that were apparent on MR imaging. The inability of CT to characterise myocardial structure and tissue characteristics as with T1/T2 (STIR) weighted MR imaging needs to be balanced against the shorter acquisition times and much larger fields of view obtained. Although, contrast CT is traditionally regarded the best non-invasive test for diagnosing coronary pathologies [18], neither modality could identify coronary thrombosis post-mortem, where MR imaging was able to confirm distinct regional infarction in the myocardium with localised hyper-intense signal on T2 weighted imaging. Technologies are currently being developed for post-mortem angiographic techniques, however these remain experimental at present [27-29].

### Study limitations

Although MR imaging has been demonstrated to have clear advantages over CT in this study, there are aspects of the technology that mitigate against ready introduction of MR equipment in even well-funded forensic pathology facilities. The equipment is expensive and usually requires extensive modifications to the autopsy building. Staff operating the equipment also require a significantly higher level of training than CT operators, and there is little expertise in interpreting autopsy MR images. Consequently, in those forensic pathology facilities where there is no access to MR expertise, this usually requires transportation of the deceased off-site, and after hours access to the imaging facility. Although about 2000 autopsies are performed at DFM per annum, and it can be expected that about 40 deaths per annum will fulfil the age and circumstances of death criteria of this study, only a minority of these deaths were able to be part of the study because of difficulties in obtaining timely consent, limited available time for scanning in the evening, and the requirement that the autopsy not be unduly delayed.

It should be noted that the positive and negative predictive value of each modality is not completely intrinsic to the imaging modality assessed, but is also influenced by the prevalence of the pathologies diagnosed, which generally occur at low levels in a population the size of Sydney, Australia. Further, as the time from death to study or true post-mortem interval is unknown in many cases of sudden death, this is likely to influence the findings of cross-sectional imaging and hence alter the diagnostic capabilities. Findings from post-mortem T2 imaging may be limited by low specificity for differentiating certain cardiac pathologies and maybe influenced by the post-mortem interval. Where there was obvious myocardial oedema on T2 weighted imaging which did not correlate with a defined coronary artery territory the imaging team made a presumptive diagnosis of myocarditis. If the widespread use of post-mortem MRI scanning were to be initiated in the future for minimally invasive autopsy, these nonspecific findings would need to be correlated with histological findings, potentially through minimally invasive biopsy of the corresponding tissue.

Finally, the comparison between whole body CT and organ dedicated MR imaging may not be regarded as an entirely fair comparison, but does reflect the “real-life” possibilities at forensic facilities required to perform this kind of investigation. Imaging at a higher field strength, such as 3 T or higher, has been shown to improve resolution and accuracy of MR imaging in the post-mortem setting in foetuses [30].

## Conclusion

We demonstrate that dedicated post-mortem MR imaging of the heart and brain to be a useful modality in the setting of sudden death in children and adults. Whole body CT was less useful for intra-cardiac pathology when compared to MR imaging, however does have a role in the diagnosis of brain and extra-cardiac lesions. In situations where for cultural reasons, for example, a traditional autopsy cannot be performed, we suggest that cross-sectional imaging can be informative as the cause of death and may have important implications for screening of first-degree family members in cardiac causes of sudden death that may be inherited.

## Competing interests

The authors declare that they have no competing interests.

## Authors’ contributions

RP performed MRI and CT studies and analysis. BG analysed data. HL and GP assisted with MRI and CT studies. LY was involved in recruitment of families. JD performed all autopsies and analysis. CS co-ordinated entire study, involved in analysis. All authors contributed to manuscript preparation. All authors read and approved the final manuscript.

## Supplementary Material

Additional file 1: Table S1 MRI techniques. Click here for file
