# Supplementary material for: Comparison of conventional autopsy and magnetic resonance imaging in determining the cause of sudden death in the young
Source: J Cardiovasc Magn Reson. 2014 Jun 19;16(1):44. doi: 10.1186/1532-429X-16-44 (PMC4067524; doi:10.1186/1532-429X-16-44)
Supplement: Additional file 1: Table S1 — MRI techniques. [file 1532-429X-16-44-S1.docx]

**Additional file 1: Table S1**
